# Novel Plaque Enriched Long Noncoding RNA in Atherosclerotic Macrophage Regulation (PELATON)

**DOI:** 10.1161/ATVBAHA.119.313430

**Published:** 2019-12-12

**Authors:** John Hung, Jessica P. Scanlon, Amira D. Mahmoud, Julie Rodor, Margaret Ballantyne, Margaux A.C. Fontaine, Lieve Temmerman, Jakub Kaczynski, Katie L. Connor, Raghu Bhushan, Erik A.L. Biessen, David E. Newby, Judith C. Sluimer, Andrew H. Baker

**Affiliations:** 1 From the Centre for Cardiovascular Science, University of Edinburgh, United Kingdom (J.H., J.P.S., A.D.M., J.R., M.B., J.K., K.L.C., R.B., D.E.N., J.C.S., A.H.B.); 2 Maastricht University Medical Center, Maastricht, the Netherlands (M.A.C.F., L.T., E.A.L.B., J.C.S., A.H.B.).

**Keywords:** atherosclerosis, macrophage, monocyte, phagocytosis, reactive oxygen species

## Abstract

Long noncoding RNAs (lncRNAs) are an emergent class of molecules with diverse functional roles, widely expressed in human physiology and disease. Although some lncRNAs have been identified in cardiovascular disease, their potential as novel targets in the prevention of atherosclerosis is unknown. We set out to discover important lncRNAs in unstable plaque and gain insight into their functional relevance.

**Approach and Results:**

Analysis of RNA sequencing previously performed on stable and unstable atherosclerotic plaque identified a panel of 47 differentially regulated lncRNAs. We focused on LINC01272, a lncRNA upregulated in unstable plaque previously detected in inflammatory bowel disease, which we termed PELATON (plaque enriched lncRNA in atherosclerotic and inflammatory bowel macrophage regulation). Here, we demonstrate that PELATON is highly monocyte- and macrophage-specific across vascular cell types, and almost entirely nuclear by cellular fractionation (90%–98%). In situ hybridization confirmed enrichment of PELATON in areas of plaque inflammation, colocalizing with macrophages around the shoulders and necrotic core of human plaque sections. Consistent with its nuclear localization, and despite containing a predicted open reading frame, PELATON did not demonstrate any protein-coding potential in vitro. Functionally, knockdown of PELATON significantly reduced phagocytosis, lipid uptake and reactive oxygen species production in high-content analysis, with a significant reduction in phagocytosis independently validated. Furthermore, CD36, a key mediator of phagocytic oxLDL (oxidized low-density lipoprotein) uptake was significantly reduced with PELATON knockdown.

**Conclusions:**

PELATON is a nuclear expressed, monocyte- and macrophage-specific lncRNA, upregulated in unstable atherosclerotic plaque. Knockdown of PELATON affects cellular functions associated with plaque progression.

## Abstract 2

Supplemental Digital Content is available in the text.

**Highlights**

- PELATON (plaque enriched lncRNA in atherosclerotic and inflammatory bowel macrophage regulation) is a novel long noncoding RNA upregulated in unstable atherosclerotic plaque.
- PELATON is highly specific to monocytes and macrophages and is expressed in the nucleus.
- PELATON is a regulator of critical macrophage functions including phagocytosis.

Cardiovascular disease remains the world’s leading cause of mortality, resulting in over 17 million deaths each year.^1^ This global epidemic is largely caused by atherosclerosis, a chronic inflammatory disease of the vasculature characterized by development of plaque in the arterial wall. Atherosclerosis accounts for >3 quarters of cardiovascular mortality in both high and low-income countries,^2,3^ and by 2020 is projected to cost the global health economy 957 billion USD per year.^4^ As the atherosclerotic plaque progresses, the vascular lumen becomes narrowed, thereby reducing delivery of blood and oxygen to the target organ, resulting in ischemia. Ultimately, advanced plaques may become unstable, carrying a risk of rupture or erosion and manifesting clinically as myocardial infarction, stroke, or critical limb ischemia, depending on anatomic location.^5,6^ Although current treatments have conferred some benefit resulting in reduced incidence and mortality of cardiovascular disease, new therapies are still needed to address the residual risk.^7^

**See accompanying editorial on page 495**

Noncoding RNAs are a class of RNA molecules involved in a wide range of pathophysiological processes and hold major potential in cardiovascular disease, with techniques to manipulate their expression constantly evolving.^6,8^ They are usually categorized by their size and function, and the best studied are microRNA and long noncoding RNA (lncRNA).^9^ MicroRNAs are 20 to 22 nucleotides in length and characteristically form a hairpin structure.^10^ They act through post-transcriptional silencing of mRNA and have particular importance in atherosclerosis.^11^ Notably, miR-126 correlates well with extent of atherosclerosis,^12,13^ and when delivered in vivo, reduces atherosclerosis burden in high-cholesterol diet mice.^14^ Additionally, miR-155 has an atheroprotective role, possibly through anti-inflammatory mechanism.^15^ In fact, numerous microRNAs are now implicated in lipid handling,^16,17^ and the behaviors of smooth muscle cell (SMC)^18,19^ and endothelial cells^20–22^ in atherosclerosis, and their use as biomarkers is extensively researched.^12,23,24^

lncRNAs are less well-studied and characterized than microRNAs, having been described more recently and are more complex in their form and functions. LncRNA are >200 nucleotides in length, can form complex secondary structures, and have a range of functions which can impact on gene regulation, such as acting as guides, sponges, or decoys.^25,26^

Recently, roles for lncRNAs in cardiovascular health have been emerging. A study of lncRNAs in 414 patients with myocardial infarction detected increases in *aHIF*, *KCNQ1OT1*, *MALAT1*, and *MIAT*, whereas *ANRIL* was decreased compared with controls.^27^ Similarly, *LIPCAR* is an important lncRNA that is highly expressed in, and predictive of future outcome following myocardial infarction.^28^ Furthermore, the lncRNA *meXis*, which is critical in cholesterol efflux in macrophages, is important in many stages in the pathogenesis of cardiovascular diseases.^29^ Although several such lncRNAs correlate with atherosclerosis disease burden,^30–32^ and others appear to be important in vascular cell behavior,^33,34^ there is no evidence to-date of a lncRNA which regulates or controls plaque instability.

We hypothesized that lncRNAs are dynamically involved in unstable atherosclerotic plaque pathology and aimed to discover and characterize novel lncRNAs, which represent feasible targets for new therapies, based on publicly available RNA sequencing databases.

## Materials and Methods

The data that support the findings of this study are available from the corresponding author upon reasonable request.

### RNA Sequencing Analysis

For sample and library preparation, see Gene Expression Omnibus database: GSE120521. Data were analyzed using RSEM and DESeq2 packages to derive fragments per kilobase million, fold change, and *P* values. Filtering was performed using Microsoft Excel for Mac, version 16.^35^

### Carotid Artery Plaque Samples for RNA

Excised carotid plaque tissue was harvested from patients undergoing carotid endarterectomy following acute neurovascular event. Local ethical approval was obtained via the NHS Lothian BioResource Tissue Governance committee (15/ES/0094). As described previously in Mahmoud et al,^35^ plaques were assessed macroscopically for dissection into stable and unstable sections. Areas of plaque rupture with associated intraplaque hemorrhage were considered unstable, and relatively healthy adjacent sections taken to be stable. Samples were stored in RNA later (Sigma-Aldrich) for 30 to 60 minutes, before snap freezing and storage at −80°C, until use for downstream validation of RNA sequencing candidates. Patient characteristics are described below (Table 1).

**Table 1.**
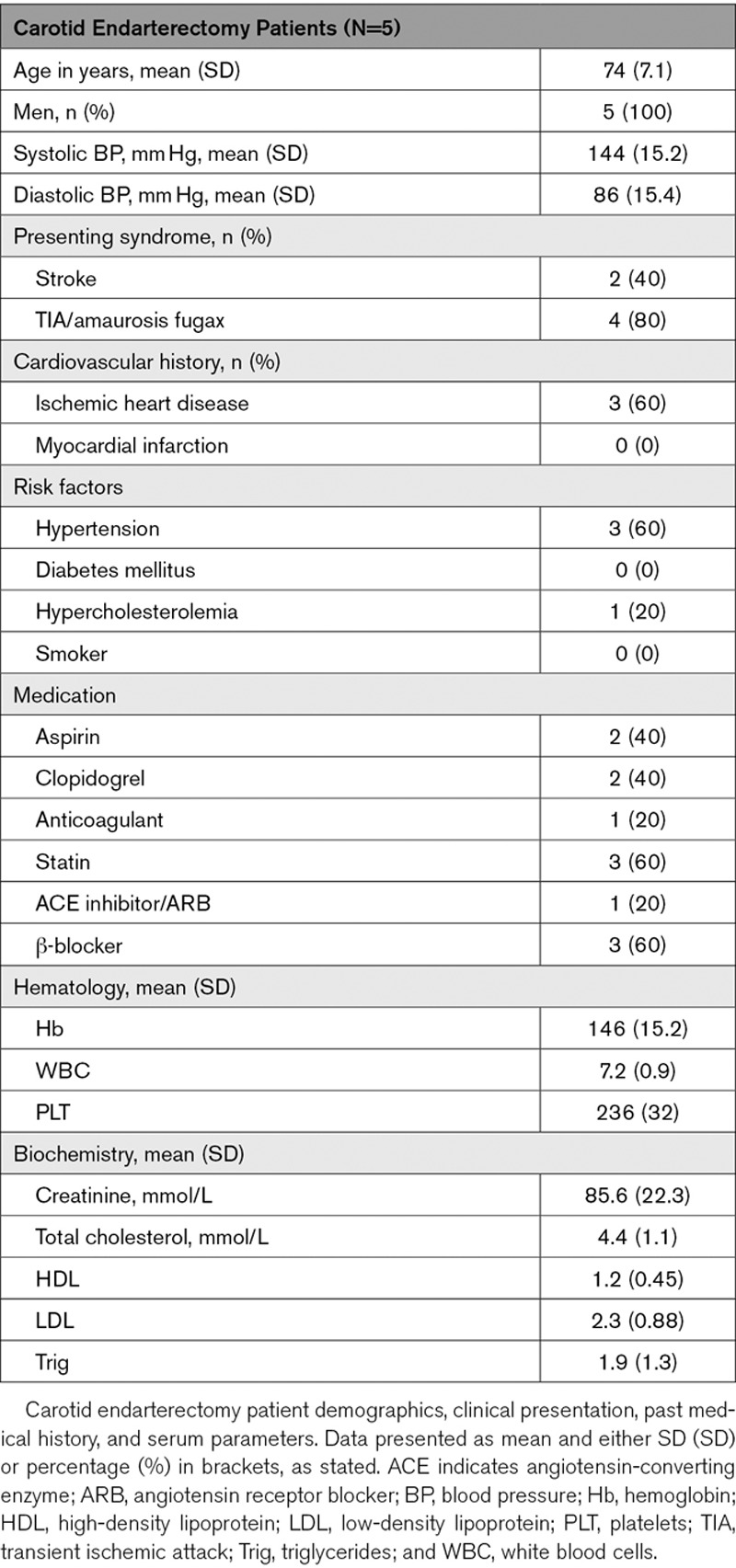
Carotid Endarterectomy Patient Characteristics

### Normal Aortic Samples for RNA Analysis

Aortic tissue was harvested from patients undergoing coronary bypass grafting, with no known aortic disease. Local ethical approval was obtained via the NHS Lothian BioResource Tissue Governance committee (15/ES/0094). Punch biopsy samples taken from grafting sites were stored in RNA later (Sigma-Aldrich), for 30 to 60 minutes, before being snap-frozen and stored at −80°C until use.

### Human Carotid Plaque Sections for In Situ Hybridization/Immunohistochemistry

Human atherosclerotic plaque samples were obtained from patients undergoing carotid endarterectomy. The tissue was part of the Maastricht Pathology Tissue Collection, and collection, storage, and use of tissue and patient data were performed in agreement with the Dutch Code for Proper Secondary Use of Human Tissue, the Declaration of Helsinki and was approved by the local Medical Ethical Committee (protocol number 16-4-181). Immediately after resection, each atheroma was divided into parallel segments of 5 mm. Formalin-fixed segments were stained with hematoxylin-eosin and according to Virmani et al,^5^ fibrous cap atheroma with or without intraplaque hemorrhage, termed stable or unstable respectively, used for in situ hybridization/immunohistochemistry. Patient characteristics were described previously.^36^

### Cell Culture

Peripheral blood mononuclear cells were isolated from whole blood of anonymized healthy control subjects, for use in fractionation RNA–fluorescent in situ hybridization (FISH), phagocytosis, and efferocytosis assays, with local ethical approval (15/HV/013). All studies were approved by East and West Scotland Research Ethics Committees, and all experiments were conducted according to the principles expressed in the Declaration of Helsinki.Leucosep porous membrane separation tubes (Greiner Bio-One GmbH, Germany) were used to isolate peripheral blood mononuclear cells, and monocytes were isolated by positive selection performed with lyophilized CD14 microbeads (Miltenyi Biotech, Gladbach, Germany). Peripheral blood mononuclear cells utilized in high-content analysis were isolated by Ficoll-Paque gradient, using buffy coat samples from Uniklinik RWTH Aachen, Germany.

For differentiation to macrophages, monocytes were cultured for 1 week at 37°C, 5% CO_2_, and stimulated with 10 ng/mL M-CSF (macrophage colony-stimulating factor; Immunotools, Friesoythe, Germany) in RPMI 1640 Glutamax (Life Technologies, the Netherlands, Europe) supplemented with 10% FBS (Gibco/Thermo Fisher, United Kingdom) and 1% penicillin/streptomycin (Gibco), with one medium change after 4 days of culture. SMCs and endothelial cells included in cell expression panel were cultured using supplemented SMC Growth Medium 2 (PromoCell, Heidelberg, Germany) and endothelial cell growth medium (EGM-2 BulletKit; Lonza, Basel, Switzerland) respectively, at 37°C, 5% CO_2_.

### GapmeR Knockdown

For selective knockdown of PELATON (plaque enriched lncRNA in atherosclerotic and inflammatory bowel macrophage regulation), GapmeRs were designed using a sequence common to all isoforms (Table IV in the online-only Data Supplement) (Exiqon, Denmark). GapmeRs were resuspended with RNase free water to a stock concentration of 10 μM and frozen at −20°C until use. Macrophages were transfected with Lipofectamine RNAiMax (Thermo Fisher Scientific) with GapmeR concentration of 20 nM for 6 hours, followed by quiescence for 18 hours.

### Human Tissue Panel

A commercially available human tissue panel, Human MTC Panel I (Takara clontech), was used to characterize expression of PELATON.

### Macrophage Polarization

Macrophages were polarized into 4 different subtypes, M1, M2a, M2b, and MoxLDL (foam cells), achieved by incubation for 24 hours at 37°C with the relevant stimuli for each; IFN (interferon) γ 20 ng/mL and lipopolysaccharide 10 ng/mL for M1; IL (interleukin)-4 for M2a at 50 ng/mL; IL-10 at 10 ng/mL for M2c and human oxLDL (oxidized low-density lipoprotein) at 50 μg/mL for MoxLDL (Table VII in the online-only Data Supplement). Polarization was confirmed by quantitative real-time polymerase chain reaction for subtype-specific markers (M1, CD68; M2a, CD200R; M2b, CD163; MoxLDL, iNOS [inducible nitric oxide synthase])

### RNA Isolation

Total RNA was isolated from human tissue and cultured cells using the miRNEasy kit (Qiagen). Tissue disruption was performed in 700 µL of Qiazol for each sample using TissueLyser II (Qiagen) for normal aortic samples and pestle and mortar in liquid nitrogen for carotid plaque samples. As per standard protocol, chloroform was added for separation of RNA fraction, which was precipitated in alcohol, and washed on minicolumns before elution in RNase free water. RNA quantity and purity was assessed using Nanodrop 1000 spectrophotometer (Thermo Scientific, Paisley, United Kingdom), confirming 260/280 ratio of 1.6 to 2.2 for all samples. Samples were stored at −80°C until required.

### Gene Expression Analysis

For quantitative gene expression analysis, cDNA was prepared from total RNA using the Multiscribe Reverse Transcriptase kit (Life Technologies, Paisley, United Kingdom). Quantitative real-time polymerase chain reaction was performed using SYBR green (Life Technologies) and custom PCR primers (Eurofins MWG, Ebersberg, Germany) or Taqman Master Mix (Applied Biosystems) with Taqman probes (primer and probe sequences; Tables I and II in the online-only Data Supplement). UBC (ubiquitin C) was used as housekeeping gene for normalization. Relative quantifications were calculated by using the 2-ΔΔCT method.^37^ When undetermined, a cycle threshold of 40 was used arbitrarily.

### Cellular Fractionation for PELATON Localization

Fractionation of monocytes and macrophages was carried out using the Ambion PARIS kit (Thermo Fisher, United Kingdom), as per the manufacturer’s guidelines. Cells were initially treated with cell fractionation buffer, resulting in a nuclear and cytoplasmic fraction. Nuclear material was further subjected to cell disruption buffer and then both fractions processed for RNA isolation using a column purification method.

### Cloning and In Vitro Translation

Open reading frame sequences from PELATON and LINC00948 were incorporated into pcDNA 3.1 (+) vectors (Geneart, Thermo Fisher), with Kozak sequence upstream, and hemagglutinin tag downstream and flanking restriction enzyme sites (Table III in the online-only Data Supplement). Translation potential was assessed using the PURExpress In Vitro Protein Synthesis Kit (New England Biolabs, MA).

### Western Blot

Protein lysates were prepared in Novex Tricine sodium dodecylsulfate (SDS) sample buffer (Thermo Fisher Scientific, MA) and dithiothreitol, incubated at 85°C, centrifuged briefly, and loaded onto a Novex 10% to 20% Tris-Glycine Mini Gel, alongside a the Spectra low range multicolor protein ladder (1.7–40 kDa range). Electrophoresis was performed at 120 V, 300A, for 1 hour, in Novex Tris-Glycine SDS Running Buffer. Membrane transfer was carried out in a Mini Blot transfer module at 20 V, 300A for 1 hour in Novex Tris-Glycine Transfer Buffer. Membranes were incubated overnight at 4°C with antihemagglutinin-tag (ab9110, 1/1000; Abcam). After washing, membranes were incubated with the appropriate Licor IRDye 680 secondary antibody (1:10 000) at room temperature for 1 hour. Following additional washing, protein levels were visualized via Licor.

### High-Content Analysis

For all assays primary human monocytes were seeded at a density of 75 to 100 000 cells per well in Corning Falcon 96-Well Imaging Microplates (Fisher Scientific). In the initial high-content analysis, 7-day differentiation into macrophages was performed, and cells were then transfected with LNA (locked nucleic acid) GapmeR targeting PELATON, as described above. Functional assays were then carried out and are described below. In each case Hoechst nuclear staining was used to identify nuclei, and images were acquired using BD Pathway 855 High Content Bioimager (BD Biosciences, CA). Nine images were taken of each individual well. Using Attovision software, stitched images were corrected for background variation, and cell segmentation (based on Hoechst signal) was carried out using appropriate threshold, watershed and object size inclusion and exclusion criteria. Data were further analyzed using DIVA software (BD Biosciences) to enable quantification of output parameters.

Apoptosis of macrophages was first assessed at 24 hours post-transfection (baseline) and after a further 24 hours treatment with 300 nM staurosporine (Sigma, Dorset, United Kingdom) for chemical induction of apoptosis. Apoptosis was detected by binding of Annexin-V-OG (locally prepared, courtesy of C. Reutelingsperger MUMC) at 2.5 ng/mL, incubated for 15 minutes at 37°C in binding buffer. Images were acquired, and apoptotic cells expressed as a proportion of total cells.

Phagocytosis was assessed by incubation with pHrodo Red Zymosan Bioparticles (Life Technologies) at 5 μL in 150 μL per well for 1 hour. Cells shown to have positive uptake of the particles were considered positive and expressed as a percentage of total cells in each well. For validation experiments, the same protocol was used, but images acquired with the Operetta High-Content Imaging System (Perkin-ElmerOH) and image analysis was performed using Columbus (Perkin-Elmer, OH) software. Image analysis produced an image that pseudo labeled cells positive (green) and negative (red) for bead uptake to allow for clear visual representation.

Lipid uptake was assessed by incubation with a mixture of 0.8 μg oxLDL (derived locally) and 0.2 μg Topfluor (Avanti Polar Lipids, AL) for 3 hours. Cells with uptake of Topfluor and oxLDL were expressed as a percentage of total cells in each well.

Mitochondrial stress was induced using 1.2 µM staurosporine for 2 hours. Mitotracker mitochondrial stain (Thermo Fisher, United Kingdom) was then added at 250 nM and cells incubated for 30 minutes. Reduced mitochondrial membrane potentials result in less uptake of Mitotracker and so a lower signal intensity. Relative average intensity was compared between wells.

Reactive oxygen species (ROS) production was stimulated using 10 µM menadione (Sigma, Dorset, United Kingdom) for 30 minutes, and ROS were detected with 20 µM dichlorofluorescin diacetate (Invitrogen, CA). ROS-positive cells were expressed as a percentage of total cells per well.

### Efferocytosis Assay

Monocytes were differentiated to macrophages and transfected with LNA GapmeR, as described previously. Jurkat cells (JC; Supplier Maastricht) were induced to undergo apoptosis by incubation with 20 nM Staurosporine for 1.5 hours at 37°C, then labeled with Calcein (Thermo Fisher). The macrophages were incubated with the JC for 1 hour and washed thoroughly. Macrophages were then detached on ice with 0.05% EDTA in PBS and run on an Attune NxT Flow Cytometer to quantify the percentage of cells which have taken up the Calcein labeled JC (fluorescein isothiocyanate [FITC]+ cells) with analysis performed on FlowJo software. Apoptosis of JC was confirmed with Annexin V.

### In Situ Hybridization + Immunohistochemistry

For in situ hybridization, formalin-fixed, paraffin-embedded tissue sections were firstly deparaffinized and rehydrated by washing in Xylene for 5 minutes, followed by sequential washes in 100%, 96%, and 70% ethanol, respectively, for 5 minutes each, then washed in water, followed by PBS for 5 minutes. The microRNA buffer set (Qiagen, 339450) was then used, providing proteinase K for tissue digestion (incubated 1:1000 at 37°C for 4 minutes) and hybridization buffer, which was used to dilute the in situ hybridization probes (Table V in the online-only Data Supplement) to 25 nM. The in situ hybridization probes were incubated on the sections overnight at 55°C, sealed using coverslips and rubber glue to prevent drying out. The sections were washed with 5× saline-sodium citrate buffer at 55°C and room temperature to increase hybridization specificity. Subsequently, the sections were washed with PBS and incubated with digoxigenin wash and block buffer set (11585762001; Roche) for 1 hour, which was subsequently used to dilute anti-digoxigenin-AP (alkaline phosphatase; 11093274910; Roche) at 1:500 and left for 1 hour at room temperature. After washing with tris-buffered saline and Tween, NBT/BCIP AP tablets (11697471001; Roche) were used for detection to visualize probe in the presence of levamisole to reduce background staining (Vector Laboratories, Peterborough, United Kingdom). Detection occurred at ≈3 hours. For immunohistochemistry costaining, slides were incubated in 0.3% hydrogen peroxide for 15 minutes, washed with tris-buffered saline and Tween 0.1% BSA, before incubation in 5% goat serum for 1 hour. Antibodies were then added (Table VI in the online-only Data Supplement) for 30 minutes, before washes with tris-buffered saline, and incubation with anti–mouse-HRP Brightvision (Immunologic, the Netherlands) for 30 minutes. After tris-buffered saline washes, Polydetector HRP green kit (Bio SB, CA), 1 drop in 2 mL was added to tissues and allowed to develop for 3 minutes. After rinsing with water, tissues were counterstained with nuclear fast red (Sigma-Aldrich), before further washing. Rehydration was done with sequential washes of 70%, 96%, and 100% ethanol, respectively, followed by xylene and mounting with Pertex.

### RNA–Fluorescent In Situ Hybridization

Custom RNA-FISH probe sets were generated to the full sequence of PELATON (Thermo Fisher Scientific). Monocytes were differentiated to macrophages as previously described, grown on 16-mm coverslips for 1 week, before being washed in PBS and fixed in 4% paraformaldehyde. RNA-FISH was performed according to manufacturer’s instructions (ViewRNA cell FISH, ThermoFisher Scientific). Briefly, the coverslips were permeabilized using detergent QS and digested in protease solution at 1:4000, then incubated with a probe sets at 1:100 (PELATON, SNORD3 [small nucleolar RNA, C/D box 3A] and UBC). The coverslips were then subsequently incubated with preamplifier, and amplifier, before finally being incubated with the label probe, which provides the fluorescent signal. Coverslips were then mounted onto glass slides using Prolong Gold Antifade Mounting Medium with DAPI (4′,6-diamidino-2-phenylindole; Vector Laboratories).

### Microscopy

An Axioscan slidescanner (ZEISS) was used to image both in situ and immunofluorescence, using Zen software (ZEISS). All settings for the Axioscan and software were optimized and then maintained for each set of experiments so that sections can be compared accurately. RNA-FISH was imaged using the Andor Revolution XDi spinning disk confocal microscope.

### Pseudo Fluorescent Image Analysis

Bright-field images for PELATON (Purple in situ staining) and CD68/aSMA (alpha smooth muscle actin; green immunohistochemistry staining) in human plaque were converted into pseudo fluorescent images using Image J Software. Images were opened in Image J, and the color deconvolution tool used, selecting regions of interest for each stain (Purple in situ staining, green immunohistochemistry staining, and pink nuclear red counterstain), generating RGB (red, green, blue) values for each color and splitting the images into these components. The RGB values generated for each color were kept consistent for further analysis across all samples. Once the images were split into the 3 colors, the nuclear stain was discounted so that the in situ and immunohistochemistry staining could be seen clearly. These were then inverted and given pseudo colors of red and green, respectively, and the resulting images merged to create a dual fluorescent image.

### Statistical Analysis

Statistical analyses were performed according to figure legends using Graphpad Prism version 8.0. Data are expressed as mean±SD. Normality of data was assessed using the Shapiro-Wilks test. Statistical difference between 2 groups was assessed using 2-tailed unpaired or paired *t* test for parametric data and Mann-Whitney or Wilcoxon-matched pairs signed-rank tests for nonpaired and paired nonparametric data, respectively. One-way ANOVA±multiple comparisons were used for ≥3 groups. Post hoc testing with Dunnett test has been performed as appropriate. Statistical significance is indicated when *P* value of <0.05 (*P<0.05, **P<0.01, ***P<0.001, ****P<0.0001).

### Graphical Image Construction

Graphical images were generated using basic components from Elsevier Medical Art.

## Results

### Identification of Dysregulated LncRNAs in RNA Sequencing of Stable Versus Unstable Plaque

**Figure 1.**
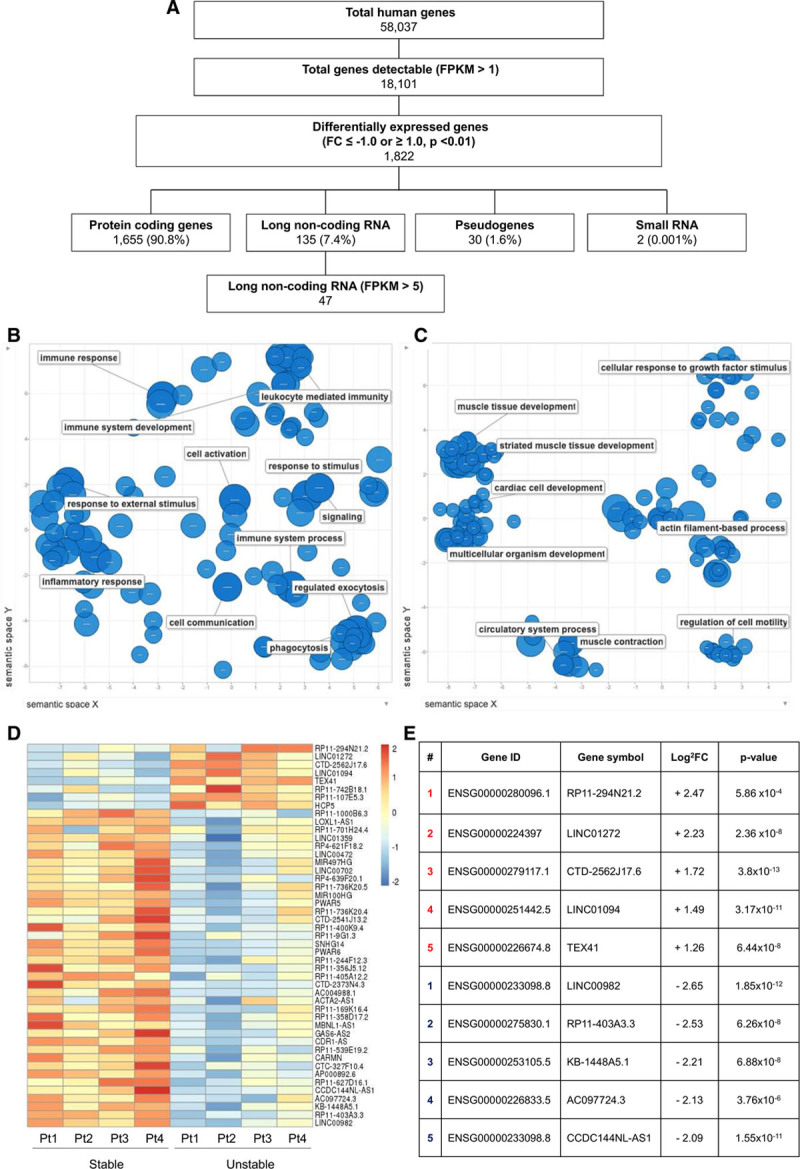
**Selection of long noncoding RNA (lncRNA) candidates from RNA sequencing in stable vs unstable carotid plaque.** **A**, Filtering strategy applied to derive a shortlist of 47 significantly dysregulated lncRNAs. Gene Ontology term enrichment analysis of (**B**) upregulated and (**C**) downregulated protein-coding genes. **D**, Heatmap showing differentially expressed lncRNAs within 4 patient samples (as row *Z* score of the Log2 [fragments per kilobase million (FPKM)+1]). **E**, Top 5 lncRNAs significantly upregulated and downregulated from candidate panel. FC indicates fold change.

To identify lncRNAs relevant to plaque stability, we utilized RNA sequencing data from stable versus unstable human carotid endarterectomy specimens previously generated in our group, with 50 million read depth (Gene Expression Omnibus database: GSE120521^35^). From a total of 58 037 genes annotated in the human genome (GRCh38), 18 101 genes were detectable across both groups. Of these, 1822 were considered to be differentially expressed once stringent filtering criteria of log2 fold change ≤ −1.0 or ≥ 1.0 and *P*<0.01 were applied. Protein-coding genes accounted for the majority 1655 (90.8%), the remainder comprised of 135 (7.4%) lncRNAs, 30 (1.6%) pseudogenes, and 2 (0.001%) small RNAs (Figure 1A). For further validation of the dataset, the panel of dysregulated protein-coding genes was analyzed for Gene Ontology term enrichment. The top 200 terms for upregulated coding genes corresponded largely with immune system processes and inflammation (Figure 1B), whereas downregulated genes were involved mainly in muscle system processes as expected (Figure 1C). Further expression filtering of the lncRNAs using fragments per kilobase million threshold of >5 subsequently identified 47 candidates, 8 upregulated and 39 downregulated (Figure 1D). The top 5 upregulated and downregulated candidate lncRNAs are shown (Figure 1E).

### PELATON Is a Nuclear, Monocyte/Macrophage-Specific Transcript

**Figure 2.**
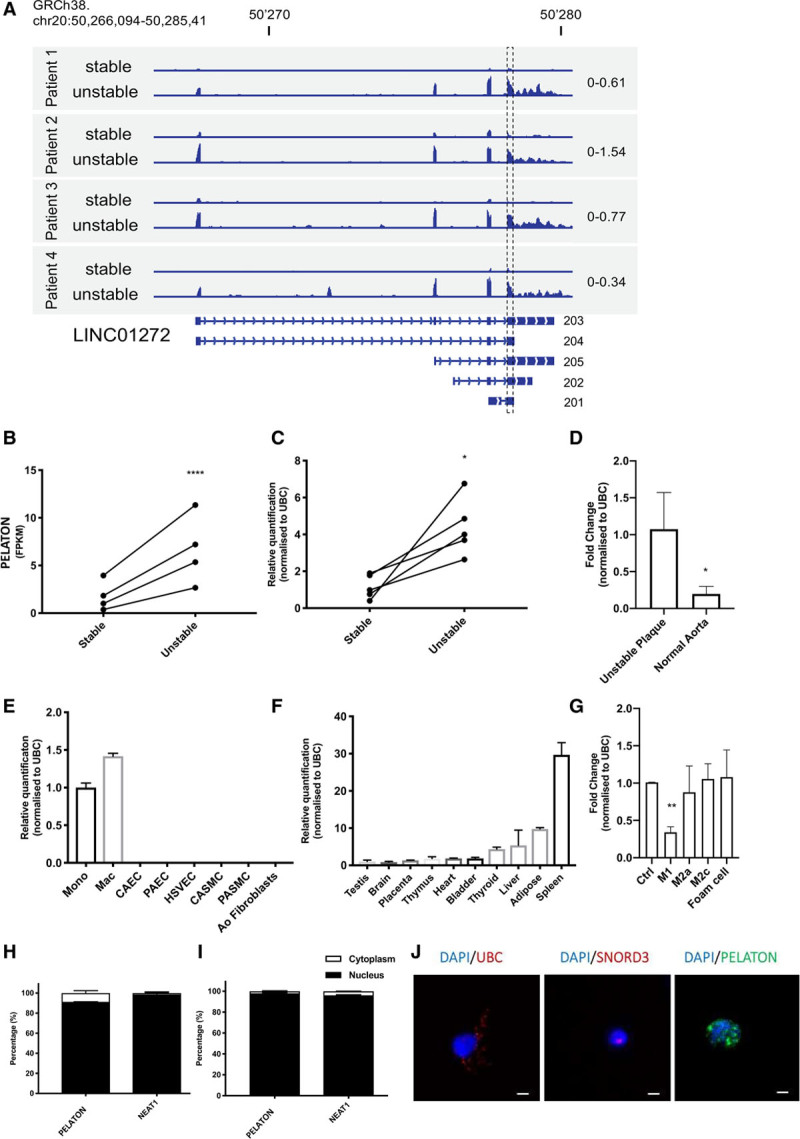
**Characterization of PELATON (plaque enriched lncRNA in atherosclerotic and inflammatory bowel macrophage regulation).** **A**, Relative read coverage of PELATON in unstable vs stable plaque from RNA sequencing, with corresponding annotated exonic structure below. The graph displays normalized read count using the same scale between stable and unstable samples for each patient (scale indicated on the right side). GapmeRs and primers were designed using a sequence common to all 5 isoforms, at the start of the final exon (dashed box). **B**, PELATON expression (as fragments per kilobase million) in stable vs unstable plaque based on the RNA sequencing analysis. The *P* value of 2.36×10^−8^ was obtained using DESeq2 based on the raw read count. **C**, Relative quantification of PELATON expression determined by quantitative real-time polymerase chain reaction (qRT-PCR) in additional samples of stable and unstable plaque obtained by qRT-PCR (*P*=0.02, *t* test, paired, 2-tailed). **D**, qRT-PCR of PELATON in expression normal aortic samples compared with unstable plaque. PELATON expression determined by qRT-PCR across (**E**) panel of vascular cell types (**F**) a panel of human tissues. **G**, PELATON expression across a panel of macrophage phenotypes (Ctrl [control macrophages], M1, M2a, M2c, foam cells). Cellular fractionation by qRT-PCR demonstrates cellular localization of PELATON in monocytes (**H**) and macrophages (**I**), using NEAT1 as nuclear control (n=3 technical replicates). **J**, RNA–fluorescent in situ hybridization in monocyte-derived macrophages of PELATON. Cytoplasmic/nuclear (UBC [ubiquitin C]) and nuclear (SNORD3 [small nucleolar RNA, C/D box 3A]) markers used as controls. Scale bar represents 5 µm. Statistical analysis by Mann-Whitney test and paired *t* test. Ao indicates aortic; CAEC, coronary artery endothelial cells; CASMC, coronary artery smooth muscle cells; DAPI, 4′,6-diamidino-2-phenylindole; HSVEC, human saphenous vein endothelial cells; Mac, macrophages; Mono, monocytes; NEAT1, nuclear paraspeckle assembly transcript1; PAEC, pulmonary artery endothelial cells; and PASMC, pulmonary artery smooth muscle cells.

The read profiles of the top differentially regulated lncRNAs were assessed to validate RNA sequencing data. This analysis revealed that the top upregulated lncRNA, RP11-294N21.2, was a false positive, as read coverage extends outside the annotation, and thus likely corresponds to background signal emanating from the nearby intron of MAPRE2 (microtubule-associated protein RP/EB family member 2) and was therefore excluded from further analysis (Figure I in the online-only Data Supplement). The second most upregulated lncRNA, LINC01272, was then chosen for validation and further investigation. We named LINC01272 PELATON (for PELATON), and will be subsequently referred to as such throughout this article. The read profile of PELATON is consistent with the upregulation in unstable plaque and shows the expression of a 4 exon-transcript, corresponding to the longest annotated isoform (203, ENST0000425497.5; Figure 2A). Each isoform shares a common region at the start of the final exon, which was subsequently used for primer and GapmeR design (dashed box, Figure 2A). From the RNA sequencing dataset, PELATON expression was 4.6-fold higher (log2 fold change 2.2) in the unstable condition (*P*=2.36×10^-8^, Figure 2B), and this differential expression was further confirmed in a quantitative real-time polymerase chain reaction validation set of N=5 independently samples carotid plaque samples (*P*=0.02, Figure 2C). Additionally, normal aortic tissue, without atherosclerosis, had significantly reduced PELATON expression when compared with unstable plaque (*P*=0.05, Figure 2D).

To determine the potential relevance of PELATON to plaque development, its cell specificity and cellular localization was assessed. A panel of relevant cell types, including immune cells, SMCs, and endothelial cells of multiple origins, demonstrated that PELATON expression is very high in monocytes and macrophages but barely expressed by other common vascular cell types (Figure 2E). A panel of healthy human tissues demonstrated highest expression of PELATON in spleen, adipose tissue, and liver (Figure 2F), all macrophage-rich tissues, consistent with the cell panel findings. Macrophages were differentiated into different subtypes (M1, M2a, M2c, and MoxLDL), and their polarization confirmed by modulation of appropriate markers in the expected direction and fold change (Figure II in the online-only Data Supplement). PELATON expression was unaltered across most subtypes but was significantly reduced in M1 macrophages, suggesting that PELATON may have a subtype-specific functional role in macrophages (Figure 2G). Inflammatory markers IL-6, IL-1, IL-1β, and TNF (tumor necrosis factor) α were, however, unchanged (Figure III in the online-only Data Supplement). Cellular fractionation experiments in both monocytes and monocyte-derived macrophages revealed that PELATON was localized predominantly within the nucleus (91% and 98%, respectively; Figure 2H and 2I). This data was further corroborated by RNA-FISH for PELATON alongside cytoplasmic/nuclear (UBC) and nuclear only (SNORD3) markers, which confirmed the nuclear localization of PELATON in monocyte-derived macrophages (Figure 2J, additional replicates Figure IV in the online-only Data Supplement).

### PELATON Localizes to Necrotic-, Inflammatory-, and Macrophage-Rich Areas of Atherosclerotic Plaque

**Figure 3.**
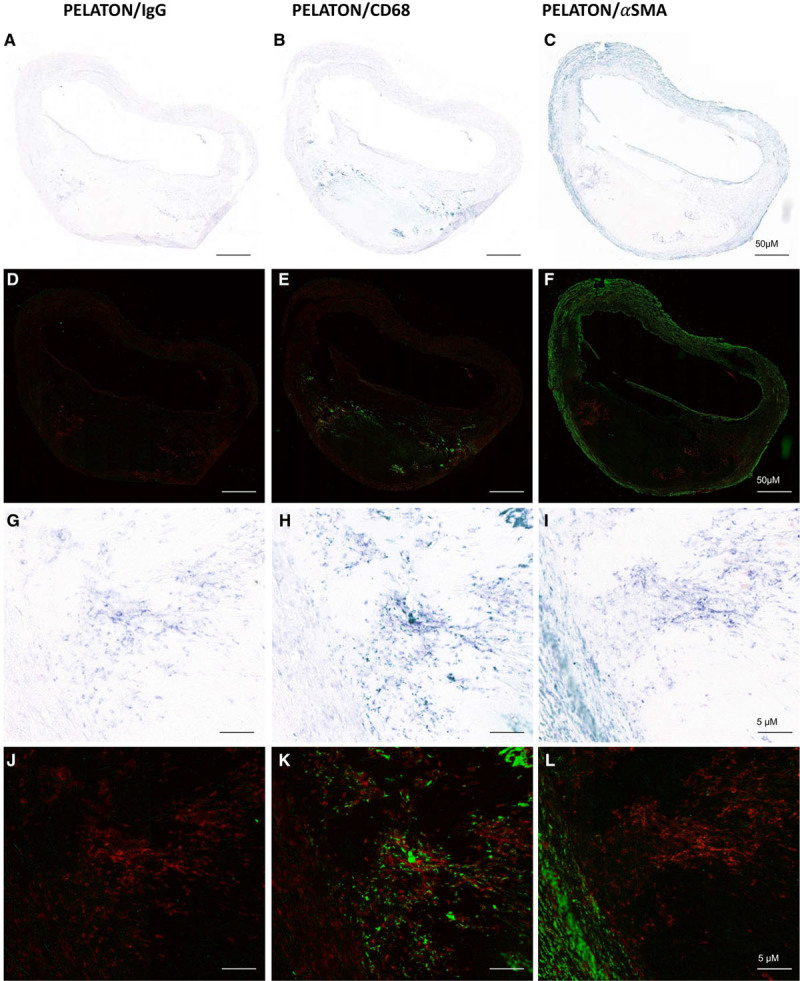
**In situ hybridization and immunohistochemistry in atherosclerotic plaque with pseudo fluorescent images.** PELATON (plaque enriched lncRNA in atherosclerotic and inflammatory bowel macrophage regulation) expression (purple **A–C**, **G–I**, Red **D–F**, **J–L**) is enriched alongside the necrotic core and plaque shoulders. PELATON colocalized with CD68 (green; **B**, **E**, **H**, **K**), but not with αSMA (alpha smooth muscle actin; green) (**C**, **F**, **I**, **J**). Pseudo fluorescent images (**D–F**, **J–L**) were produced with Image J, to allow for clearer visualization of PELATON and CD68/aSMA staining and colocalization. n=3 biological replicates Scale bars represent 50 µm in large plaque images (**A–F**) and 5 µm in magnified images (**G–L**).

To further confirm the localization and cell-type expression of PELATON within the plaque, we undertook in situ hybridization with simultaneous immunostaining of macrophages (CD68) or SMCs (αSMA) in atherosclerotic plaque excised at carotid endarterectomy from N=3 patients. Nonspecific in situ hybridization probe (Scramble) and IgG immunohistochemistry control antibodies were negative, confirming probe and antibody specificity (Figure V in the online-only Data Supplement). Pseudo fluorescent images were produced in Image J to allow for better visualization of costaining (Figures 3D through 3F and 3J through 3L). PELATON localizes to regions adjacent to the necrotic core and plaque shoulder (Figure 3A through 3F). CD68 consistently colocalized with PELATON on same tissue staining (Figure 3B, 3E, 3H, and 3K), and there was only a slight PELATON signal in the αSMA-positive SMC layers (Figure 3C, 3F, 3I, and 3J). These data, therefore, provide in vivo confirmation PELATON expression described in vitro in of the cell panel, as PELATON is predominately expressed in lesional macrophages. This thereby suggests a potential functional role for PELATON in atherogenesis.

### PELATON Is a LncRNA Not a Protein-Coding Gene

**Figure 4.**
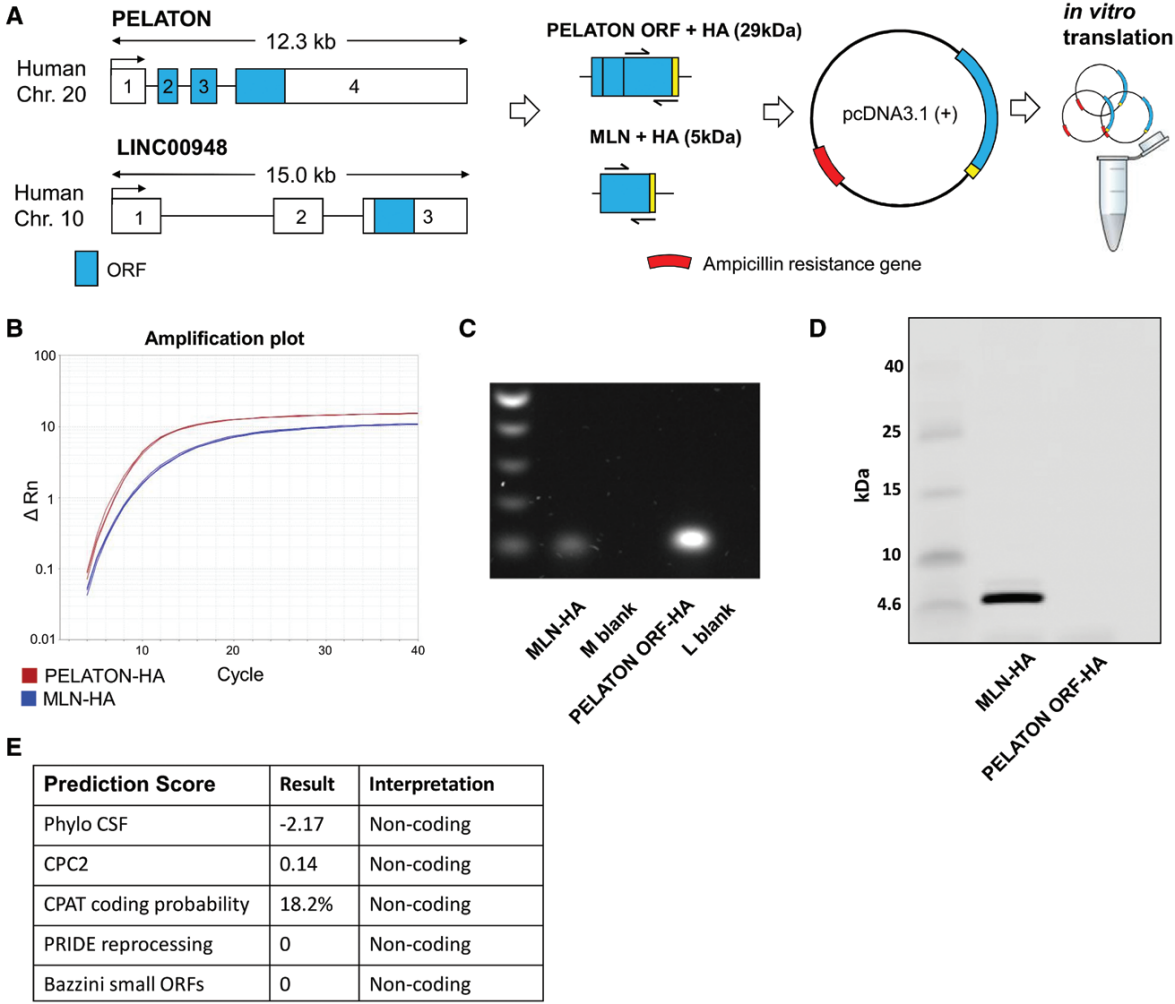
**PELATON (plaque enriched lncRNA in atherosclerotic and inflammatory bowel macrophage regulation) does not encode a micropeptide.** **A**, PELATON predicted open reading frame (ORF; blue) appended with a hemagglutinin (HA) tag (yellow) was inserted into pcDNA 3.1(+) plasmid for in vitro translation assay. LINC00948 was used as a positive control, known to encode the micropeptide MLN (myoregulin). RNA expression using customized primers spanning ORF/HA boundary confirming transcription into RNA from plasmids by (**B**) quantitative real-time polymerase chain reaction (PCR) and (**C**) agarose gel electrophoresis of PCR products. **D**, Western blot using HA-antibody to detect tagged peptide produced by in vitro translation. **E**, Prediction scores estimating probability of protein-coding potential. M blank+L blank denotes quantitative real-time PCR negative controls for MLN-HA+PELATON ORF-HA, respectively. CPAT indicates coding potential assessment tool; CPC2, coding potential calculator version 2; CSF, codon substituion frequencies; and PRIDE, proteomics identification database.

Although PELATON was identified as a lncRNA in our screening approach, it has been previously annotated with multiple names, including RP11-290F20.3, GCRL1 (gastric cancer–related lncRNA 1)^38^ and SMIM-25 (small membrane integral protein 25). To clarify if PELATON has any protein-coding potential, the putative open reading frame NP_001265584, as predicted by Refseq, was inserted into a pcDNA 3.1(+) vector with a hemagglutinin tag and cloned for in vitro translation assay (Figure 4A). Sequencing of the resultant plasmid confirmed the intended base pair sequence was present, along with the transcription start site and standard Kozak sequence upstream. The plasmid was then inserted into a proprietary in vitro translation kit, with all the cellular components necessary for translation contained. Successful transcription of mRNA was detected for both PELATON and our positive control by quantitative real-time polymerase chain reaction (Figure 4B and 4C; using primers across the ORF sequence and hemagglutinin tag boundary), but PELATON transcript was not translated to a peptide, confirmed by Western blotting. A positive control, LINC00948 known to encode the micropeptide MLN (myoregulin),^39^ indeed encoded a peptide of the expected size of 5 kDa (Figure 4D). In addition, bioinformatic predictions of protein-coding probability universally indicate noncoding is most likely (Figure 4E).^40–44^ Together, this data supports the noncoding nature of PELATON, further confirming it as a bona fide lncRNA.

### PELATON Does Not Act in *cis*

**Figure 5.**
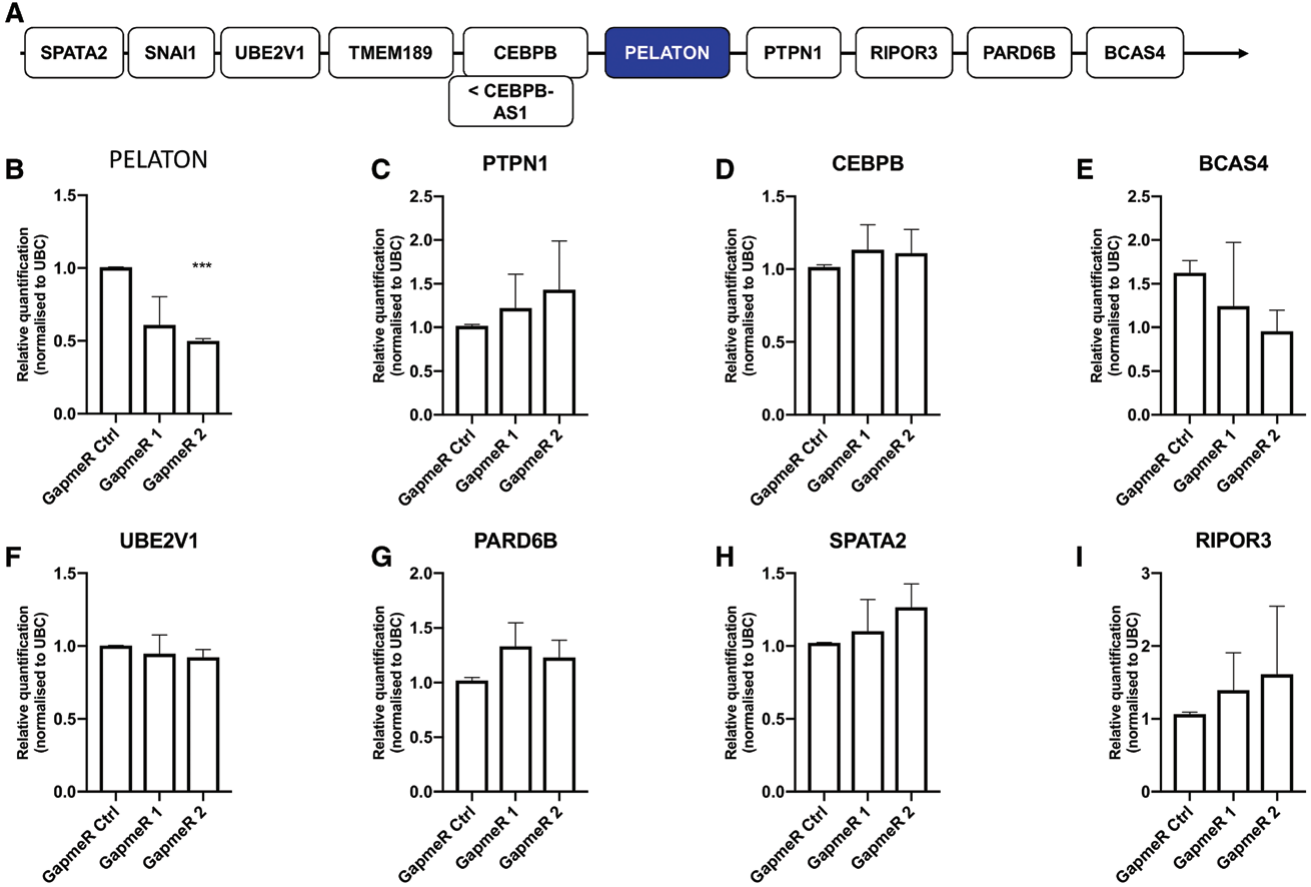
**Effect of PELATON (plaque enriched lncRNA in atherosclerotic and inflammatory bowel macrophage regulation) knockdown on nearby genes a.** **A**, Neighboring genes upstream and downstream to PELATON within a 1 megabase. **B–I**, Effects of GapmeR knockdown on the expression of neighboring protein-coding genes by quantitative real-time polymerase chain reaction in monocyte-derived macrophages. n=3 biological replicates, statistical analysis by 1-way ANOVA.

As several lncRNAs are known to act on nearby genes at the same locus (in cis), neighboring genes on chromosome 20 were further analyzed for regulation when PELATON levels were reduced by interference (Figure 5A).^45^ The protein-coding gene *CEBPB*, occupying a locus 74 kb upstream from PELATON, is implicated in the human immune response, and in macrophage function, and was, therefore, a potential candidate for PELATON regulation.^46^ However, none of the genes in a 1 megabase span around PELATON were observed to be dysregulated in monocyte-derived macrophages under PELATON knockdown conditions (Figure 5B through 5I).

### Knockdown of PELATON in Macrophages Impairs Macrophage Function In Vitro

**Figure 6.**
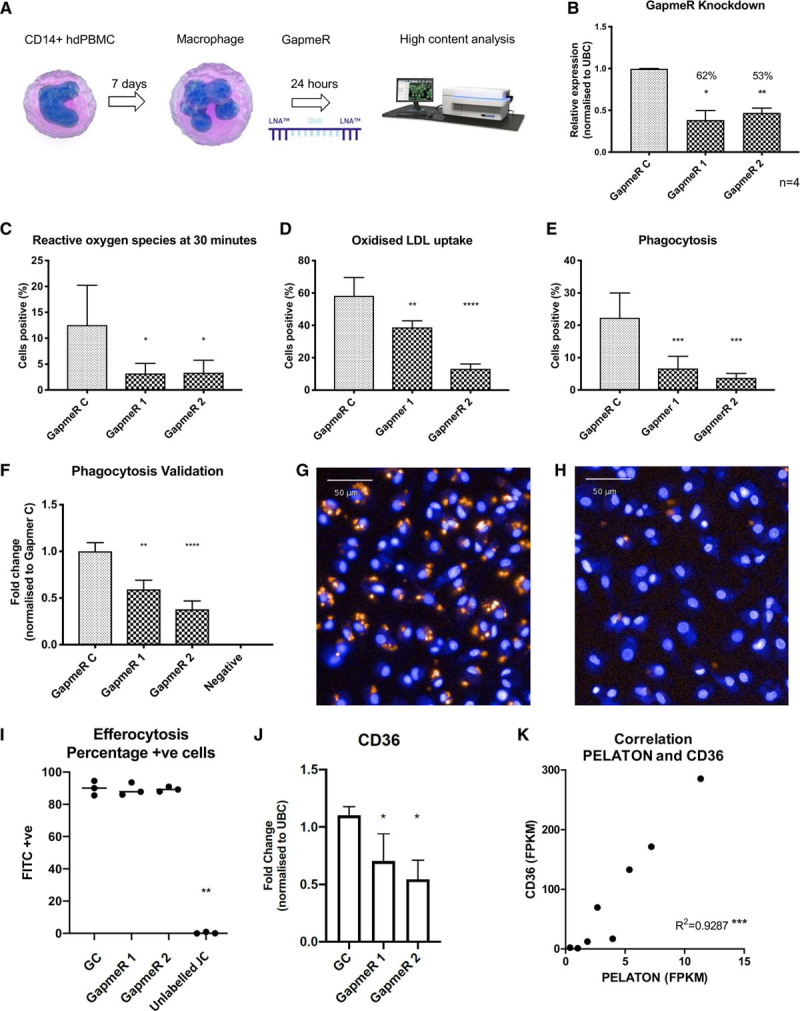
**Effects of PELATON (plaque enriched lncRNA in atherosclerotic and inflammatory bowel macrophage regulation) knockdown on macrophage function.** **A**, Schematic of high-content analysis workflow. **B**, GapmeR knockdown of PELATON in monocyte-derived macrophages. In high-content analysis, PELATON knockdown has a significant effect on (**C**) reactive oxygen species production after menadione induction, (**D**) oxidized LDL (low-density lipoprotein) uptake, and (**E**) phagocytosis. All HCA assays (**C–K**) performed in 5 wells per condition, 9 images per well, and n=2, pooled biological replicates. **F**, Validation of phagocytosis data assessed via pHrodo Zymosan particle uptake (orange beads), (3 biological replicates on different days, 11 to 14 wells per condition, 9 to 21 images per well assessed). Hoechst nuclear stain allows for cell detection. Representative images of (**G**) GapmeR control and (**H**) PELATON knockdown wells. **I**, Efferocytosis quantification following PELATON knockdown, calculated by the percentage of cells that have taken up Calcein labeled jurkat cells that had undergone apoptosis. **J**, Effect of PELATON knockdown on expression levels of CD36. **K**, Correlation of PELATON with CD36 expression in plaque based on fragments per kilobase million (FPKM) value from RNA sequencing data. Statistical analysis by 1-way ANOVA and multiple comparisons. FITC+ indicates fluorescein isothiocyanate positive cells; GC, gapmer control; HCA, high content analysis; hdPBMC, human-derived peripheral blood mononuclear cells; JC, Jurkat cells; and UBC, ubiquitin C.

To assess the role of PELATON in macrophage behavior and function, a GapmeR knockdown approach was used (Figure 6A). Two GapmeRs were designed to target a common sequence in all PELATON isoforms, with both GapmeRs achieving a reduction in expression of 53% to 62% that were subsequently used for all assays (Figure 6B). High-content image analysis established that PELATON knockdown did not change macrophage cell area, cell perimeter, mitochondrial stress, baseline apoptosis, apoptosis stimulated by staurosporine, or baseline ROS production (Figure VI in the online-only Data Supplement). However, there was a reduction in menadione stimulated ROS production at 30 minutes (Figure 6C), uptake of oxLDL (Figure 6D), and phagocytosis (Figure 6E). Further validation in an independent assay of phagocytosis was carried out via pHrodo Zymosan particle uptake, modeling phagocytosis of large particles, such as protein aggregates or erythrocytes (Figure 6F through 6H). The pHrodo Zymosan particles become fluorescent once within the phagosome, seen in Figure 6G and 6H. These images were analyzed with Columbus software, to provide automatic quantification, detecting the presence of macrophages positive (green) and negative (red) for bead uptake (Figure VII in the online-only Data Supplement). This assay confirmed the findings of Figure 6E, with PELATON knockdown causing a consistent reduction in phagocytosis (Figure 6F). Conversely, phagocytosis did not affect PELATON levels (Figure VIII in the online-only Data Supplement). To assess if other forms of endocytosis were affected, we performed an assay of efferocytosis, the uptake of apoptotic macrophages, using fluorescently labeled JC which had undergone apoptosis. Under these conditions, PELATON knockdown did not affect efferocytosis (Figure 6I, Figure IX in the online-only Data Supplement), unlike phagocytosis. CD36 is a key receptor in oxLDL uptake and phagocytosis, both of which were altered by PELATON knockdown. To determine if CD36 could be involved in the mechanism of action for PELATON, its expression in control and PELATON knockdown conditions was assessed. This demonstrated that PELATON knockdown results in a 39% reduction in CD36 expression (Figure 6J). Furthermore, we interrogated the RNA sequencing results from the initial patient samples of stable and unstable carotid artery plaque (Figure 1) for CD36 expression and determined there was a strong positive correlation (R^2^=0.9287, *P*=0.0001) between PELATON and CD36 (Figure 6K). This strengthens the findings from the knockdown experiment that the mechanism of PELATON action may be due to modulation of CD36. Taken together these data confirm that PELATON depletion has a functional impact on human macrophages.

## Discussion

Based on available RNA sequencing data in human carotid endarterectomy samples, we have identified and characterized the novel lncRNA PELATON, which is enriched in unstable atherosclerosis, and expressed almost entirely in the nuclear fraction of monocytes and macrophages. Depletion of PELATON in monocyte-derived macrophages in vitro markedly reduces the cell’s ability to perform phagocytosis. Phagocytosis of lipoproteins or erythrocytes is integral to plaque progression and strongly associated with development of plaque instability.^47^ Our research suggests that this lncRNA contributes further to the growing body of evidence that the noncoding genome is fundamentally involved in wide-ranging pathologies, including atherosclerotic vascular disease.

The previous paired-sample design of RNA sequencing was key in ensuring the discovery of differentially expressed lncRNAs in atherosclerotic plaque. Utilizing paired stable and unstable samples from the same patient overcomes the inherent variability in gene expression between human subjects, which may otherwise mask significant fold changes. This is particularly important in lowly expressed transcripts, such as lncRNAs, when absolute copy numbers per cell may be lower than protein-coding genes.^48^ Furthermore, a high read-depth enabled accurate alignment of reads to the most recent human genome annotation (GRCh38). Notably, lncRNAs represent a relatively small proportion of all of the differentially expressed genes (7.4%), and protein-coding genes predominate (90.8%), as would be expected in these atherogenic, proinflammatory conditions. Gene Ontology analysis of the most dysregulated protein-coding genes provides further validation of the RNA sequencing output, demonstrating an increase in terms related to inflammatory and immune processes in the unstable tissue, whereas muscle cell processes were down (Figure 1B and 1C). Though these groupings are typically broad, a reduction in the smooth muscle fibrous cap is characteristic in instability, as is enhanced infiltration by inflammatory cells.^49,50^

Given that the samples used for RNA sequencing were of a heterogeneous cellular composition, the cell-specific expression of PELATON needed to be determined. Both in vitro and in situ analysis allowed us to confirm that PELATON is highly specific to monocytes and macrophages (Figure 2E and 6) and is localized to the nucleus (Figures 2H through 2J). This specificity of expression, along with its abundant expression, suggests that PELATON is a functionally important lncRNA to the monocyte and macrophage population. Interestingly, although PELATON has been identified in several studies of other inflammatory conditions, it has never been fully characterized. In a biomarker study by Wang et al,^51^ PELATON expression was upregulated in tissue and plasma of patients with inflammatory bowel disease, and similarly Lin et al^38^ found it to be upregulated in tissue and plasma of patients with gastric cancer, As with other well-known lncRNAs, it is likely that biological significance is not limited to just one disease process, but more likely multiple, especially given the ubiquity of monocytes and macrophages in human disease.

To localize PELATON in human atherosclerotic plaques, staining of PELATON by in situ hybridization was undertaken alongside immunohistochemistry for macrophage marker CD68 and SMC marker αSMA (Figure 3). These findings confirm that PELATON is most enriched in areas where macrophages are known to aggregate, and least apparent in smooth muscle and adventitial layers, with predominantly nuclear intracellular localization. It is important to note that although colocalization with CD68 is extremely frequent, it is on occasion not exclusive. Instances where PELATON is present without CD68, are likely explained by several possibilities. Within the atherosclerotic plaque, there are heterogeneous macrophage populations, of which not all types will express CD68. Furthermore, SMCs undergoing phenotypic switching may also express macrophage markers like CD68, but not PELATON. On contrary, there may be monocytes that have not yet differentiated and thus will not express CD68 but may express PELATON.

With new genomic annotations, protein-coding potential is sometimes assigned to sites previously designated as noncoding, which is relevant in the case of several recent discoveries.^39,52^ In addition, micropeptides emanating from lncRNA transcripts may be implicated in their mechanism of action and are very likely to affect the overall function of the gene, but require experimental validation.^39,53^ In the case of PELATON, although it was historically named as LINC01272 on Ensembl, it has also been referred to as GCRL1 and further renamed as SMIM-25 based on Refseq’s prediction of a possible encoded peptide NP_001265584. Due to the critical nature of this point, we demonstrate that the putative protein-coding sequence contained within the final 2 exons of PELATON is not translated into a peptide, under the experimental conditions we used. In addition, bioinformatics tools were all uniform, predicting PELATON to be noncoding (Figure 4).

Discovery of the functional significance of novel lncRNAs is challenging given their extremely wide-ranging functions.^26^ Furthermore, atherosclerosis is a complex disease, given the numerous different cell types involved, pathophysiological pathways occurring in parallel, and the myriad of crosstalk between cells and organic compounds. LncRNAs can commonly act to affect gene in cis; however, nearby genes were unaffected by PELATON knockdown (Figure 5). Our high-content analysis approach was hence designed to screen a number of well-known macrophage functions relevant to atherosclerosis. GapmeRs were used to knockdown PELATON expression, as they are the tool of choice for nuclear transcripts.^54^ GapmeR knockdown of PELATON had no effect on general cell size (area and perimeter) nor apoptosis (Figure VI in the online-only Data Supplement). However, key atherosclerosis processes of phagocytosis, oxLDL uptake, and ROS production were markedly affected by GapmeR knockdown of PELATON (Figure 6C through 6E). Impairment of phagocytosis upon knockdown of PELATON was further validated in an independent assay (Figure 6F through 6H), whereas the specific pathway of efferocytosis was unaltered (Figure 6I, Figure IX in the online-only Data Supplement). It is particularly noteworthy that these dysregulated processes are closely linked and are all central to plaque macrophage pathophysiologies, such as foam cell development, inflammation upregulation, and the progression of the plaque to a vulnerable and unstable phenotype.

The overall effect of phagocytosis in the plaque is complex, with opposing effects at different stages. In the early plaque environment, phagocytosis may be beneficial, as macrophages are the principal phagocytes of apoptotic cells (including apoptotic macrophages) and in doing so can reduce lesion size. As the lesion progresses, the potentially helpful process of dead cell removal is by far outweighed by the negative remodeling achieved by sequestration of erythrocytes, platelets, and modified lipoproteins. Thus, any modulation of this process to attempt to reduce the rate of plaque formation would have to be tightly regulated, and understanding the precise method of action of PELATON, and how it affects these pathways, would be essential before PELATON can be targeted in a clinical setting. Dying cells and various forms of LDL (oxLDL, acetylated LDL, aggregated LDL) are recognized by receptors including CD36, CD68, SRA (scavenger receptor class A), and LOX-1 (lectin-like oxidized low-density lipoprotein receptor-1).^47^ Interestingly, CD36 expression significantly reduces with PELATON knockdown (Figure 6J), further confirming that PELATON is involved in regulating this pathway. Utilizing the RNA sequencing data from stable and unstable sections of carotid artery plaque, we were able to demonstrate a strong positive correlation of PELATON and CD36 expression (Figure 6K), backing up the in vitro data from Figure 6J. Lipid uptake transforms the macrophage into a foam cell, which ultimately undergoes necrotic or apoptotic cell death and empties its contents into the enlarging necrotic core. Macrophages and foam cells are also key producers of ROS, as well as proinflammatory cytokines and MMPs (matrix metalloproteinases), which additionally promulgate the inflammatory cascade.^55^ A lncRNA that positively regulates phagocytosis, such as PELATON, represents a potential target for reducing the rate of this process and hence plaque progression. To our knowledge, there are no such therapies in development at present. Local delivery of an anti-PELATON oligonucleotide such as a GapmeR would be preferred over systemic delivery, to avoid off-target effects. As such, coronary artery plaques are easily accessible percutaneously, and administration during angioplasty using a drug-eluting balloon, for example, would allow treatment of specific lesions.

Further mechanistic analysis of how PELATON exerts its effects on macrophage behavior is now critical, to understand how potential therapies could work, and how modulation would impact other pathways within the plaque and the organism as a whole. The finding that PELATON knockdown causes a reduction in CD36 mRNA (Figure 6K) confirms that PELATON has the ability to modulate receptors in key macrophage functions, further backing up the importance of these future investigations. Additional experiments will be expansive and beyond the scope of the current article. We conclude that PELATON represents an exciting lncRNA discovery, with biological significance in the functions of the macrophage and potential applications in treatment of inflammatory conditions such as atherosclerosis. Further work to define disease-specific functions and mechanistic interactions is now critical to take these findings forward.

**Table 2.**
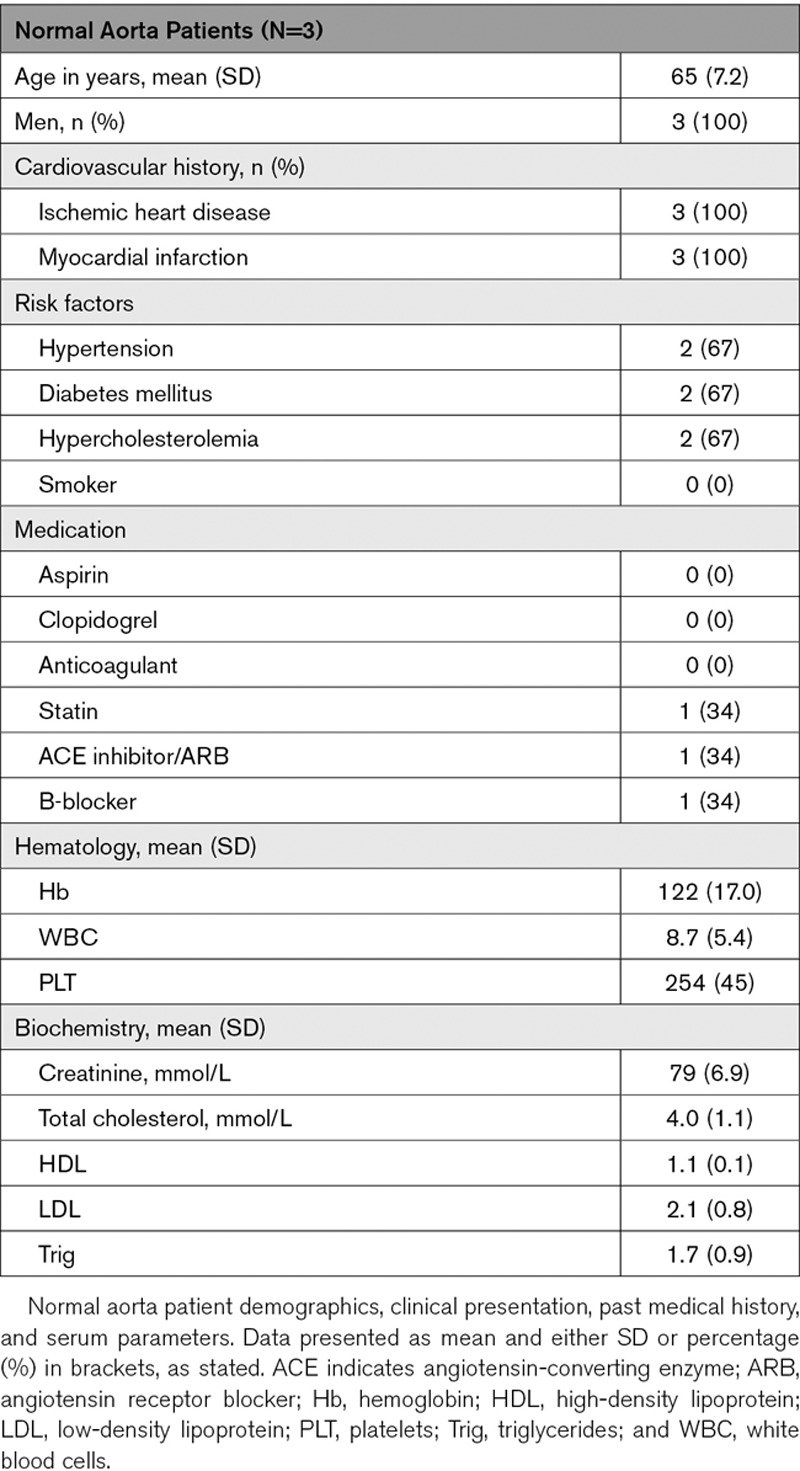
Normal Aorta Patient Characteristics

## Acknowledgments

We thank Gregor Aitchison, Yvonne Harcus, and Kathryn Newton for technical support. Flow cytometry data was generated with support from the QMRI Flow Cytometry and cell sorting facility, University of Edinburgh.

## Sources of Funding

This work is supported by the British Heart Foundation (BHF; PG/16/51/32180 and RG/14/3/30706) and the University of Edinburgh’s BHF Research Excellence Award (RE/13/3/30183 and RE/18/5/34216). A.H. Baker is supported by the British Heart Foundation Chair of Translational Cardiovascular Sciences (CH/11/2/28733) and European Research Council (EC 338991 VASCMIR).

## Disclosures

None.

## Supplementary Material


