# Supplementary figures and images for: Novel Plaque Enriched Long Noncoding RNA in Atherosclerotic Macrophage Regulation (PELATON)
Source: Arterioscler Thromb Vasc Biol. 2019 Dec 12;40(3):697–713. doi: 10.1161/ATVBAHA.119.313430 (PMC7043732; doi:10.1161/ATVBAHA.119.313430)

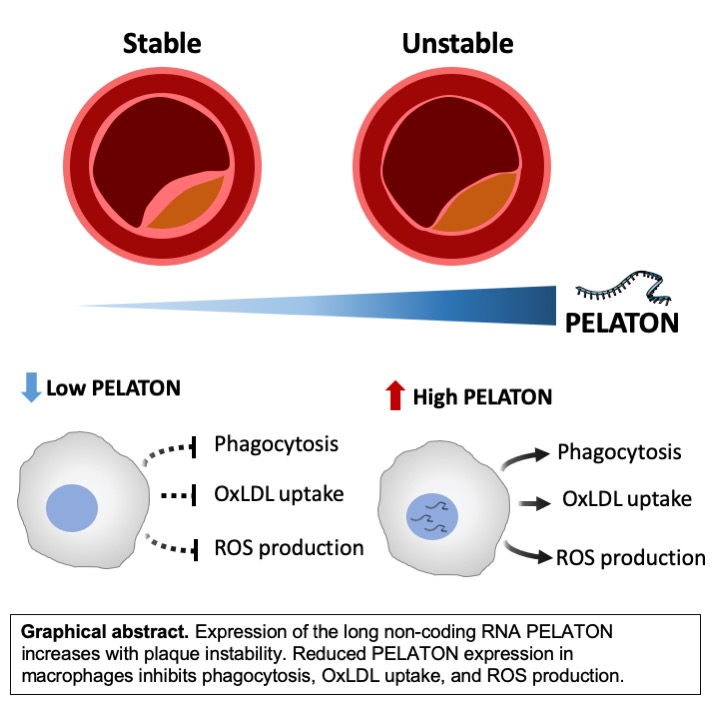

Supplement: Supplementary file 1 [file atv-40-697-s001.jpg]
