# Supplementary material for: Novel Plaque Enriched Long Noncoding RNA in Atherosclerotic Macrophage Regulation (PELATON)
Source: Arterioscler Thromb Vasc Biol. 2019 Dec 12;40(3):697–713. doi: 10.1161/ATVBAHA.119.313430 (PMC7043732; doi:10.1161/ATVBAHA.119.313430)
Supplement: Supplementary file 2 [file atv-40-697-s002.pdf]

Supplemental Material

Title:
A Novel Plaque Enriched Long Non-Coding RNA in Atherosclerotic Macrophage Regulation (PELATON)

Authors and affiliations:
J Hung[1#], JP Scanlon[1#], AD Mahmoud[1*], J Rodor[1*], M Ballantyne[1], MAC Fontaine[2], L Temmerman[2], J Kaczynski[1], KL Connor[1], R Bhushan[1,3], EAL. Biessen[2], DE Newby[1], JC Sluimer[1,2†], AH Baker[1,2†]
[1]Centre for Cardiovascular Science, University of Edinburgh, Edinburgh, UK
[2]Maastricht University Medical Center, Maastricht, The Netherlands
[3]Current affiliation, Yenepoya Research Centre, Yenepoya University, Deralakatte, Mangalore, India
[#, *, †]equal contribution

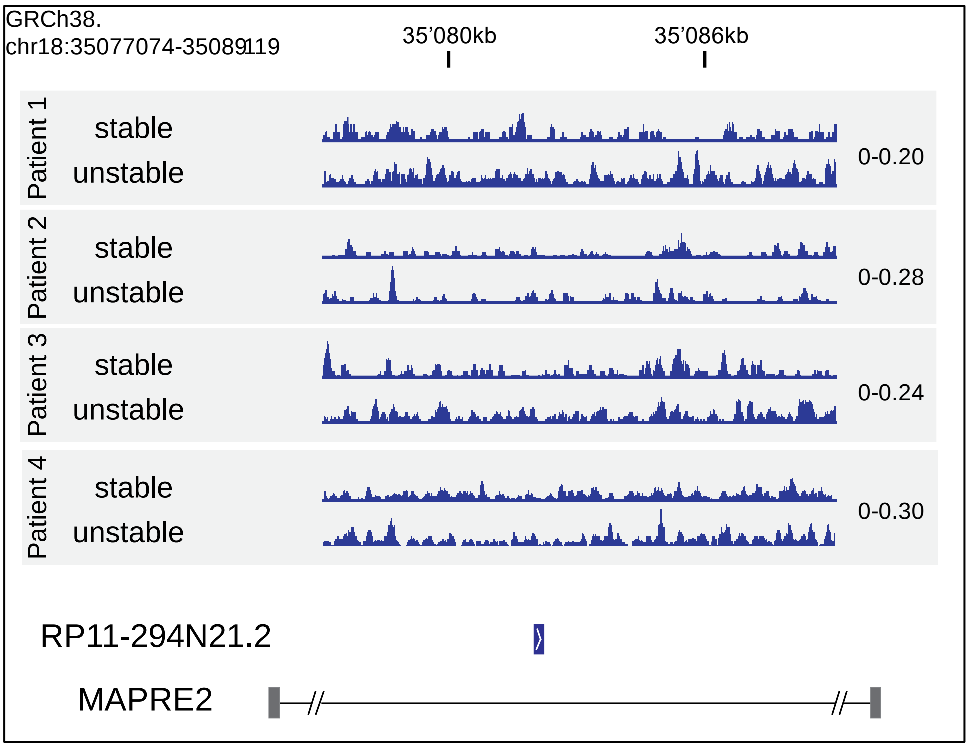

Supplementary Figure 1: RP11-294N21.2 read mapping from RNA sequencing. Reads for RP11-294N21.2 are randomly distributed, not consistent with an exonic structure and likely emanate from the nearby protein coding gene MAPRE2, and therefore represents a false positive.

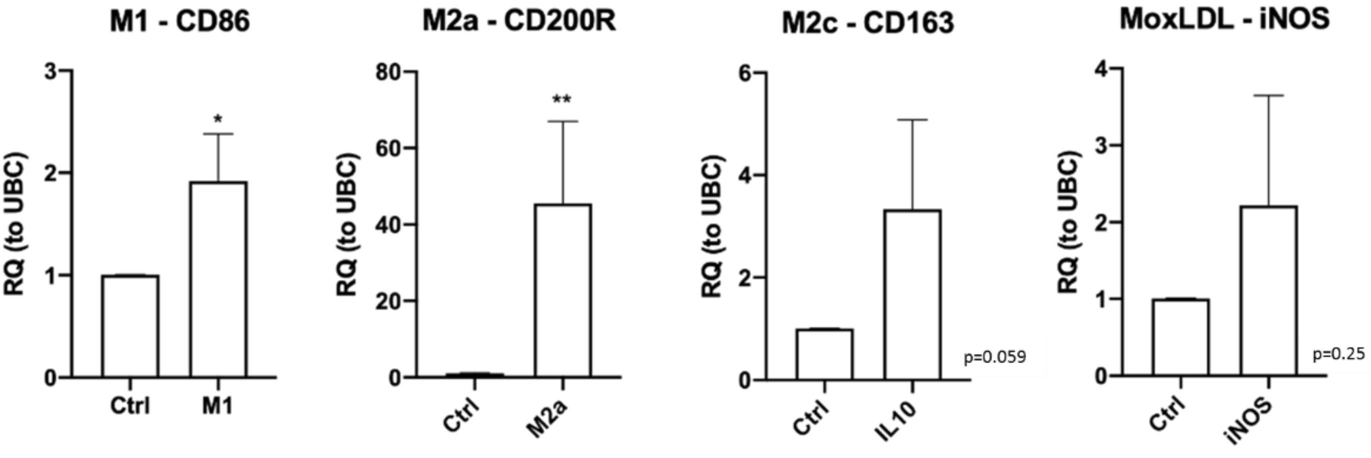

Supplementary Figure 2. Markers of macrophage polarisation. The expression of macrophage subtype specific markers was quantified by qRT-PCR on samples which had undergone phenotypic differentiation. N=3.

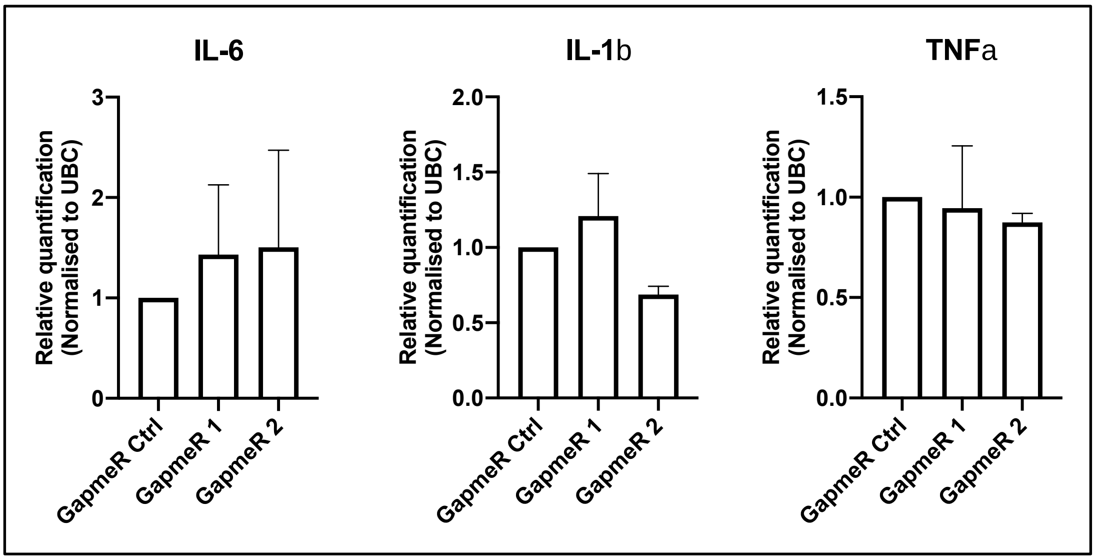

Supplementary Figure 3. Inflammatory markers in PELATON Knockdown. Expression of IL-6, IL-1β and TNFα were unchanged in cells where PELATON had been knocked down. The Kruskal-Wallis test applied for statistical significance, n=3 biological replicates.

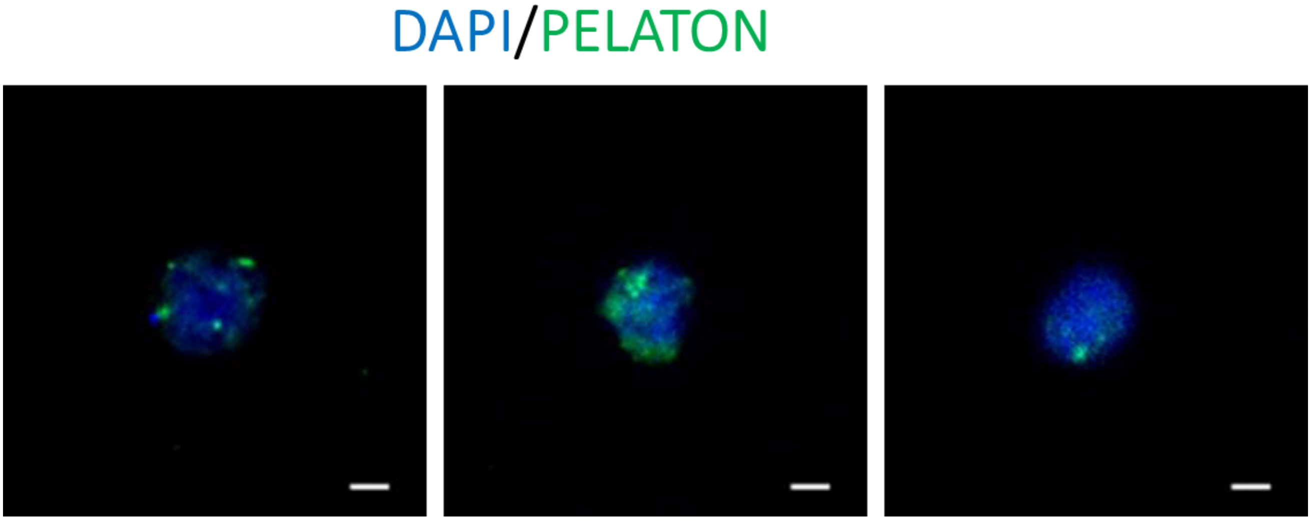

Supplementary Figure 4. PELATON localisation in monocyte derived macrophages. Localisation of PELATON (green) with DAPI nuclear costain (Blue), shown by RNA-FISH. Scale bar represents 5µm.

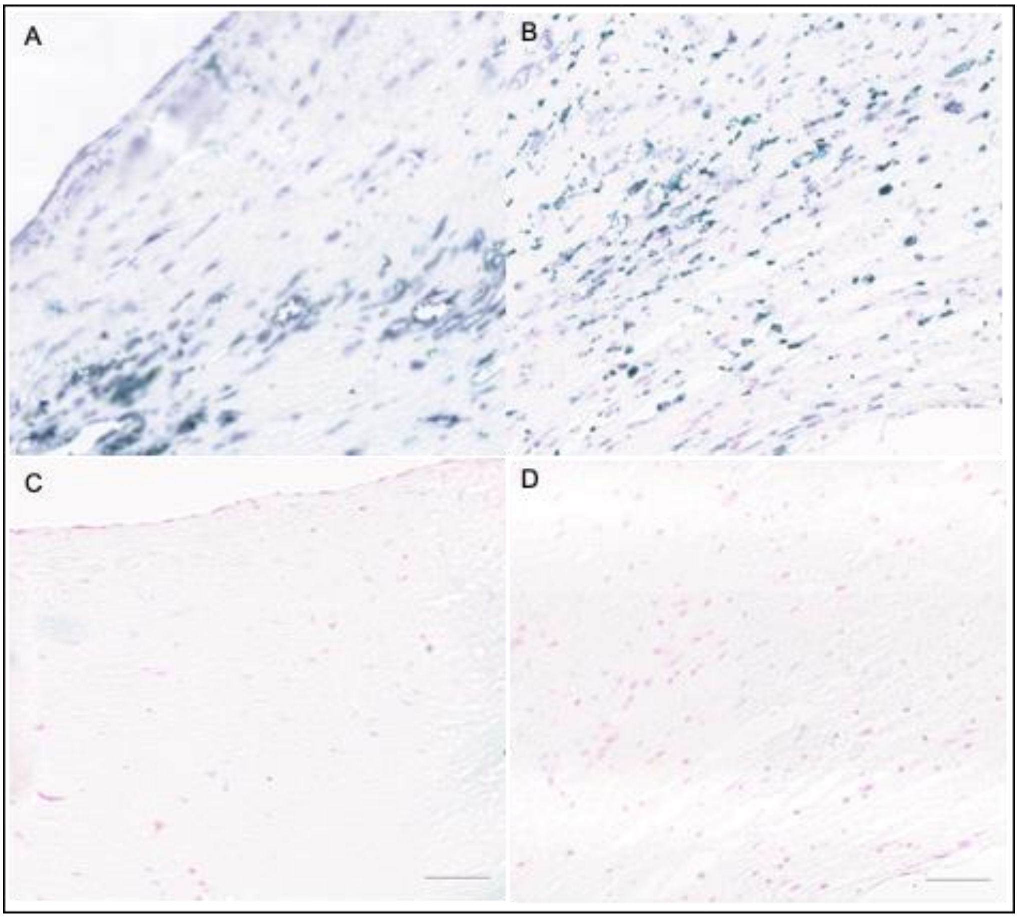

Supplementary Figure 5: In situ hybridisation in atherosclerotic plaque. (A, B) Co-localisation **of** PELATON (purple) with CD68 (green) n=2 replicates. (C, D) Corresponding serial sections of A, B stained with negative controls, scramble probe and IgG, n=2 replicates.

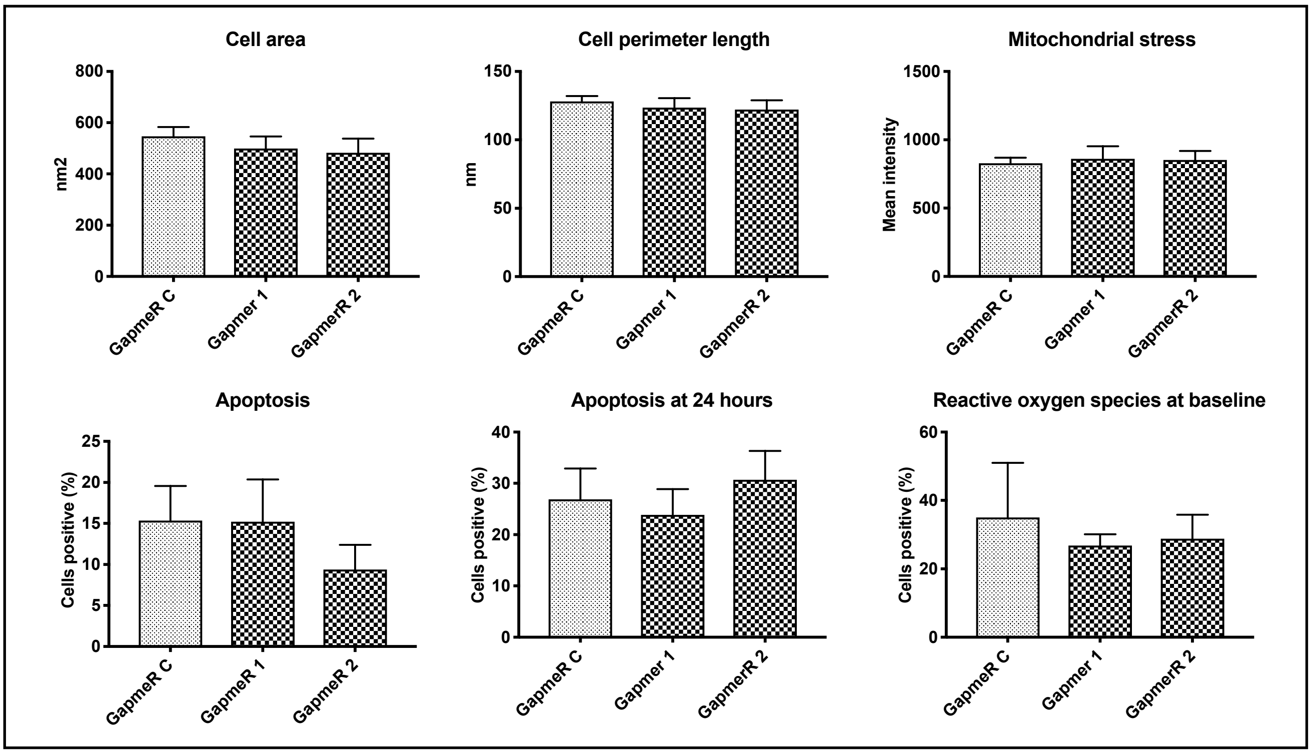

Supplementary Figure 6. Effect of PELATON knockdown on macrophage functions *in vitro*. High content analysis on the effect of PELATON GapmeR knockdown on cell area, cell perimeter length, mitochondrial stress, apoptosis, apoptosis after 24 hours of staurosporin induction, and ROS species production at baseline. All HCA assays performed in 5 wells per condition, 9 images per well, biological n=2, pooled.

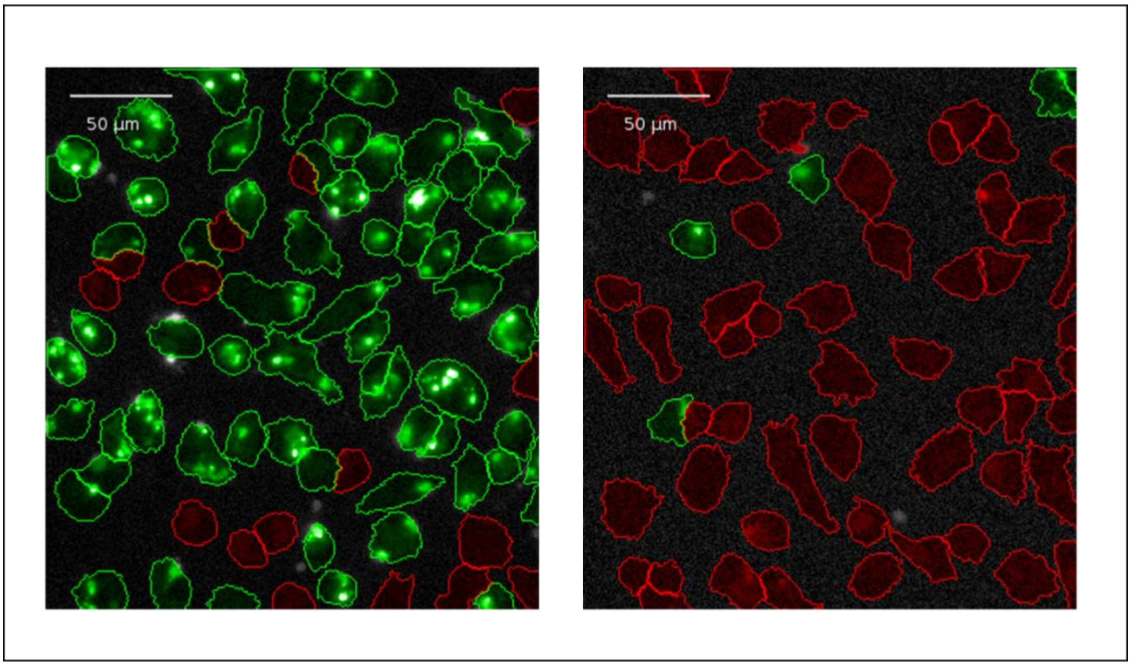

Supplementary Figure 7. Effect of PELATON knockdown on phagocytosis *in vitro*. Images showing uptake of Red Zymosan Bioparticles in GapmeR control and PELATON GapmeR knockdown wells (Figure 5G, H), were run though Coumbus Analysis Software, automatically detecting cells via Hoechst, and the presence/absence of a phagocytosed particle within them, and showing them as green (positive) or red (negative). Representative images of control well and knockdown well.

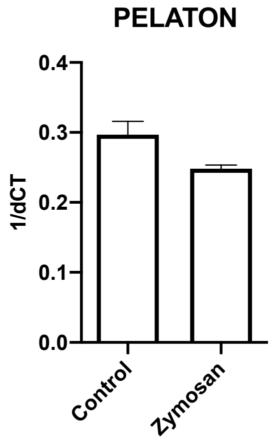

Supplementary Figure 8. Expression of PELATON in phagocytosis. PELATON expression was not significantly different in phagocytosing macrophages (Zymosan) compared with control. Wilcoxon matched pairs test for statistical significance, n=5 biological replicates.

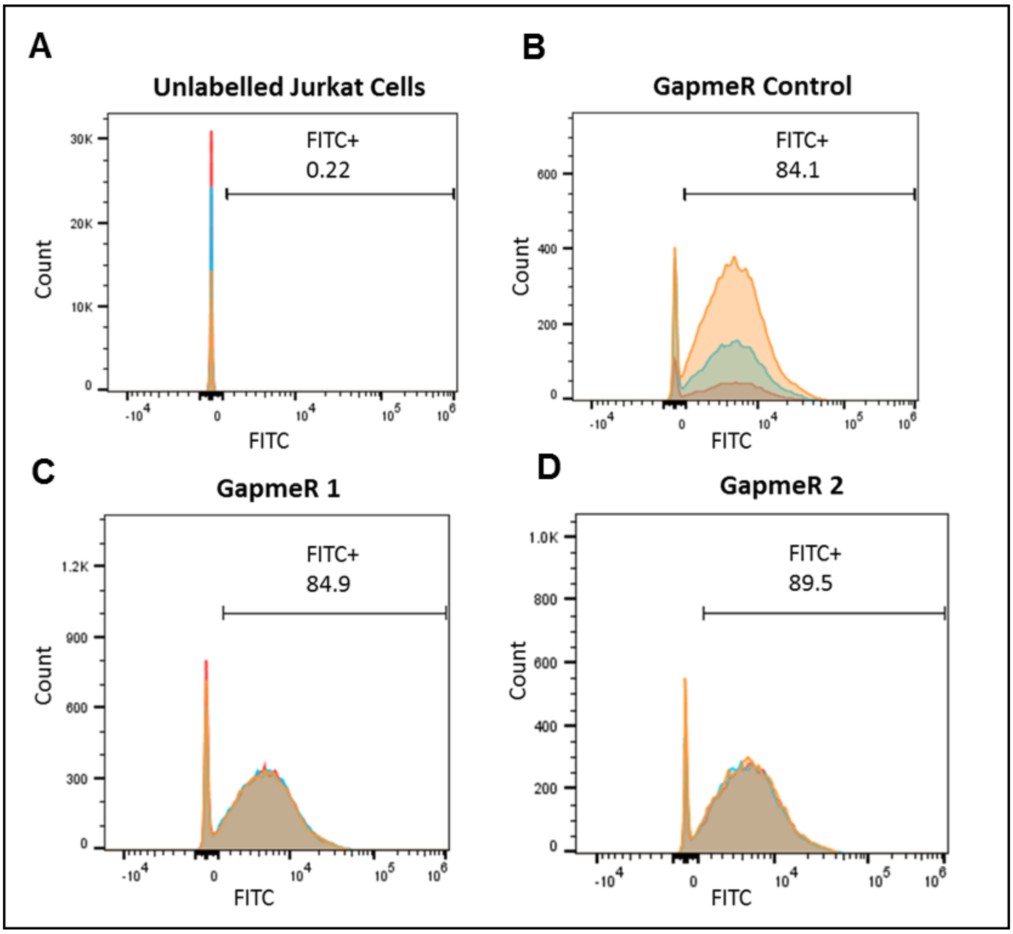

Supplementary Figure 9. The effect of PELATON knockdown on efferocytosis. FACS histogram, to show the presence of Calcein labelled (FITC+) Jurkat cells within macrophages, shown as percentage labelled in macrophages. (A) Unlabelled jurkat cells are used as a negative control. The percentage of FITC+ macrophages in (B) GapmeR control, and (C, D) PELATON specific GapmeRs,. Each plot shows an overlay of 3 replicates (orange, blue, red lines). FITC+ =Fluorescein positive cells.

Gene ID	Sequence
PELATON_Fw	CCTTCCTTCTGCCTCCACTG
PELATON_Rv	TTGCTGTGGACTGATGTGGG
NEAT1_Fw	TTGGGACAGTFFACGTGTGG
NEAT1_Rv	TCAAGTCCAGCAGAGCA
PELATON+HA_Fw	GCCTCCACTGCCACCACTGC
MLN+HA_Fw	AAGATGAAATTGTGGGAAGA
HA_only_Rv	AGCGTAATCTGGAACATCGT
BCAS4_Fw	GAGCTCGCGCTCTTCCTGAC
BCAS4_Rv	AGGGGCTGGCTCTCATTGGT
UBE2V1_Fw	TGGAGTGGTGGACCCAAGA
UBE2V1_Rv	TAACACTGTCCTTCGGGCG
PARD6B_Fw	GGGCACTATGGAGGTGAAGA
PARD6B_Rv	TCCATGGATGTCTGCATAGC
SPATA2_Fw	AGCCAGACTTTGTTGGATTTG
SPATA2_Rv	TTTACTGGCGATGTCAATAGG
RIPOR3_Fw	TCCATTGAAGAGGAGGCTCG
RIPOR3_Rv	TCCCCTCCTAAGTTCCTCCA
CD200R_Fw	TGGATGAAAAACAGATTACACAGAA
CD200R_Rv	TAATGCGATAGGAGGGCAAC
CD163_Fw	GATGTGGATCTGCACTCAAA
CD163_Rv	TCCAGAGAGAAGTCCGAATC
iNOS_Fw	CTTTGATGAGGGGACTGGGC
iNOS_Rv	ATGTTCTTCACTGTGGGGCTTG

Supplementary Table 1: SYBR Primers

Gene ID	Catalogue Number
PTPN1	Hs00942477_m1
CEBPB	Hs00942496_s1
IL-6	Hs00174131_m1
IL-1β	Hs01555410_m1
TNFα	Hs00174128_m1
CD68	Hs02836816_g1

Supplementary Table 2: Taqman Probes

Gene	Inserted sequence
PELATON	AAGCTTGGATCCCCACCATGAAGCTACTTGCCAAGGTCACGCAGCACA GTCACATCCTACTGAACATCATCCTGTTCTCTGGGTGGAATGTCACCAT CGCCCAGGTGGGGATTTTTGTGTGTTTTGTTCACTGCTGTACACCCAGC CCCCAGCACAGCGCCTGTCCAGGACAAGTGCCCAGTAAACACTTGGGA AGCAATGCAAGCGTCCTCCCAGCAGCTCCTGCAAACAGACCCCCGACC CAAGCCCTTCCTTCTGCCTCCACTGCCACCACTGCTGCTCATCTCTGCT GGCACAGAAGTCTCTTCCCTGGTCTTCCAGAAATCCCCTCTCCACACTC AGCCAGAGGGAGCTATTACCCATACGATGTTCCAGATTACGCTTAACTC GAGGATATC
LINC00948	GGATCCCCACCATGACTGGTAAAAACTGGATATTAATTTCTACTACTACT CCCAAAAGTCTAGAAGATGAAATTGTGGGAAGACTTCTAAAAATTTTGTT

	TGTTATCTTTGTTGACTTAATTTCTATTATATATGTTGTGATAACTTCTTA CCCATACGATGTTCCAGATTACGCTTAACTCGAG

Supplementary Table 3: Open reading frame sequences.Both genes were flanked with restriction enzyme digestion sites, Kozak sequence, appended with HA tag, and inserted into pcDNA 3.1 (+) vectors.

Gapmer ID	Sequence
GapmeR control	AACACGTCTATACGC
GapmeR 1	GAAGGGCTTGGGTCG
GapmeR 2	AAGGAATCCGAGGGT

Supplementary Table 4: GapmeR sequences

Probe ID	Sequence
Scramble control	GTGTAACACGTCTATACGCCCA
PELATON	TTATTCTCCAAGCAACAGAGAT

Supplementary Table 5: In-situ hybridisation probes

Antibody	Supplier	Concentration	Dilution
CD68	Dako	185mg/L	1:500
SMA	Dako	71mg/L	1:8000
IgG	Invitrogen	3000mg/L	1:4000

Supplementary Table 6: Immunohistochemistry probes

ID	Supplier	Cat no
LPS	Thermo, UK	00-4976-93
IFNγ	Immunotools	11343534
IL-4	Immunotools	11340042
IL-10	Immunotools	11340102

Supplementary Table 7: Polarisation stimuli

Major Resources Tables

Animals (in vivo studies)

Species	Vendor or Source	Background Strain	Sex
N/A	-	-	-

Animal breeding

	Species	Vendor or Source	Background Strain	Other Information
**Parent - Male**	N/A	-	-	-
**Parent - Female**	N/A	-	-	-

Antibodies

Target antigen	Vendor or Source	Catalog #	Working concentration	Lot # (preferred but not required)
CD68	Dako	M0814	1:500	-
SMA	Dako	M0851	1:8000	-
IgG	Invitrogen	10400c	1:4000	-

Cultured Cells

Name	Vendor or Source	Sex (F, M, or unknown)
N/A	-	-
